# Psychological Impact of COVID-19 on Frontline Healthcare Workers in Saudi Arabia

**DOI:** 10.7759/cureus.15300

**Published:** 2021-05-28

**Authors:** Sulafa Alqutub, Mahmoud Mahmoud, Tahani Baksh

**Affiliations:** 1 Family and Community Medicine, University of Jeddah, Jeddah, SAU; 2 Family and Community Medicine, Imam Muhammad Ibn Saud Islamic University, Riyadh, SAU; 3 General Directorate, Ministry of Health, Jeddah, SAU

**Keywords:** males, coping behavior, healthcare workers, kessler psychological distress scale, k10, covid 19

## Abstract

This study assesses the determinants and severity of psychological distress among frontline Ministry of Health workers within Saudi Arabia during the rapid acceleration phase of the coronavirus disease 2019 (COVID-19) epidemic. Moreover, we assess distress sustainability and stress-coping behaviors. We conducted an online national cross-sectional survey. The Kessler Psychological Distress Scale (k10) is a highly reliable instrument used to assess depression and anxiety. We evaluated stress-coping behavior and the persistence of the disorders. Binary logistic regression identified the sociodemographic factors related to severe distress.

The prevalence of severe psychological distress among COVID-19 frontline healthcare workers (HCWs) was 27.3%. Factors associated with severe psychological distress in multiple regression analyses were male gender (p < 0.001), working for >45 hours/week (p = 0.009), age of >40 years (p = 0.038), years of experience for more than seven years (p = 0.048), Asir region (p = 0.003), and using psychological services (p < 0.001). The prevalence of severe psychological distress was 27.3%. Factors associated with severe psychological distress in multiple regression analyses were male gender, working >45 hours/week, age, years of experience, region, and using psychological services. The results form a foundation for targeted psychological health support services at the individual and institutional levels to prevent progression to mental illness.

## Introduction

Since the beginning of the coronavirus disease 2019 (COVID-19) pandemic and with the announcement of the first confirmed case in March 2020, the Saudi Ministry of Health (MOH) and Saudi Center of Disease Control (SCDC) took the lead in the battle against the disease. According to the latest statistics for 2019, the Saudi MOH oversees 286 hospitals or 57% of the hospitals in the Kingdom of Saudi Arabia (KSA). The total number of primary healthcare centers (PHCS) is 2261. During the 2020 pandemic, 20 MOH hospitals were referral centers for COVID-19 cases across the nation, along with a variable number of quarantine facilities; these facilities dealt with suspected and/or confirmed cases of COVID-19 [1]

Frontline healthcare workers (HCWs) are workers in direct contact with suspected and/or confirmed cases within any previously mentioned facilities. Workers face considerable stress, anxiety, and depression. Hospital workers, especially in the emergency department, intensive care unit, and infectious disease ward, are at greater risk of anxiety, depression, and psychological disorders [2].

The Kessler psychological distress scale (k10) is a psychometric instrument designed as a sensitive tool to discriminate cases of serious mental illness (SMI) from non-cases using a cutoff for the range of clinically significant distress. Australian and Canadian national health interview surveys use the k10. According to the Victorian population health survey, the k10 does not determine major mental illnesses such as psychosis. The k10 uses 10 questions to measure a subject’s psychological distress over the previous one-month period. The k10 has been validated as a simple measure of anxiety, depression, and worry, or what is generally called psychological distress [3].

The Arabic k10’s reliability and validity were tested on Arabic speakers in different countries, including occupied Palestinian territories [4]. Arguments ensued about the k10 translation and culture adaptation and the difficulty of establishing cutoffs for all cultural groups in multicultural societies. However, recommendations say to use the k10 as a screening tool followed by a further clinical assessment to rule out mental illness [5].

During the COVID-19 epidemic in China, Kang et al. stated that protecting medical workers’ mental health is crucial for controlling the epidemic and protecting their health [6]. The most recently published local study describing the psychological burden among nurses in Riyadh, KSA, concluded that practicing male nurses were more likely to suffer from the psychological burden attributed to the COVID-19 pandemic [7].

This work aimed at measuring the severity of psychological distress and the contributing factors affecting the frontline moh workers, during the rapid escalation phase of the COVID-19 pandemic within KSA.

## Materials and methods

Inclusion and exclusion criteria, instrument, data collection methods, and sites

This cross-sectional survey included frontline HCWs at the Saudi MOH facilities. In addition to information on sociodemographic factors, job category, and years of experience, we used the K10. The Arabic version of the K10 was developed and used by the World Health Organization Composite International Diagnostic Interview Advisory Committee. The instrument is available in Arabic and English on the US National Comorbidity survey web page of the National Comorbidity Survey (NCS) [8]. The recently validated K10 version psychometric construct used its first six items to assess the severity of anxiety and its last four to assess the severity of depression. The anxiety scale included tiredness, nervousness, severe nervousness, restlessness, severe restlessness, and feelings toward the effort. The depression-scale items included hopelessness, depressed mood, the severity of depressive mood, and feelings of worthlessness. Each item within the K10 was based on a five-point scale from 0 (none of the time) to 5 (all of the time) and added for a total score ranging from 0 to 50. Higher scores indicated higher levels of psychological distress [9].

The cutoff point scores for K10 to differentiate between nonspecific distress and cases with SMI were pre-determined in the 2001 Victorian population health survey. The cutoff point for the scores was: 10-19 likely well, 20-24 mild disorder likely, 25-29 moderate disorder likely, and 30-50 severe disorder likely [3]. In this survey, we adopted the cutoff point scores from the Victorian population health survey.

Moreover, in addition to the K10, respondents who reported psychological distress in the past 30 days answered additional questions on the disorder’s persistence and its impact on their health and ability to perform daily routines [8]. We assessed stress-coping skills through questions on exercise, meditation, and smoking habits. Questions on psychological or psychiatric help-seeking behavior over the last 12 months determined professional mental health service use in the past year [8].

**Figure 1:**
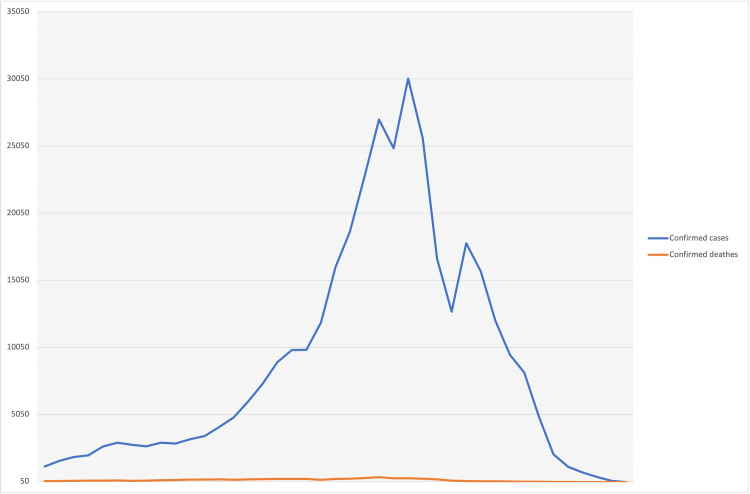
National weekly confirmed cases and deaths from the 10th-50th epidemiologic weeks of the 2020 epidemic curve in Saudi Arabia Note: data collection was performed during the 20th-26th epidemiologic weeks of 2020.

The online data collection link remained open from the 20th through the 26th week of the 2020 epidemic, a period marked by a rapid acceleration in the epidemic curve (Figure 1) [10]. The MOH intranet portal sends the data collection link containing the Arabic and English language versions.

Sample size calculation and statistical analysis

In 2016, a published study on psychological distress in Saudi Arabia among medical students using the K10 reported a psychological distress prevalence of 34% [11]. The proportion of surveyed HCWs with psychological co-morbidities was roughly 35% based on a previous study of the severe acute respiratory syndrome coronavirus (SARS) outbreak [12]. A total of 228,171 HCWs, including Saudis and non-Saudis, are in the MOH. The estimated number of frontline participants in the COVID-19 team is 30% of the total number of participants (68,451). OpenEpi (www.OpenEpi.com) info was used, and the concluded sample size was 1901 at the 95% confidence interval with a margin of error of 2.1%. We used the following equation in the sample size calculation.

n = [DEFF*Np(1-p)]/[(d2/Z21-α/2*(N-1)+p*(1-p)]. Where DEFF is the design effect, p* is the estimated proportion.

For this analysis, the K10 score was divided into two groups: severe psychological disorder and nonsevere psychological disorder. HCWs with scores of 30-50 were part of the severe psychological disorder category, and HCWs with scores of 10-29 were part of the nonsevere psychological disorder category.

Frequency distributions were used to describe categorical variables. The mean and standard deviation reported central tendency measures. Differences between groups were analyzed using the chi-square test for categorical variables and the independent t-test for continuous outcome variables. Significance (P) was set at 0.05. All the significant variables in the univariate analysis were entered into a multiple regression model to identify the independent predictors of severe psychological distress. Analysis was performed with the Statistical Package for the Social Sciences (SPSS) (IBM Corp., Version 21.0. Armonk, NY).

The K10’s reliability was tested using Cronbach’s alpha.

## Results

Sociodemographic and work-related characteristics

Two-thousand ninety-four (2,094) of 2,499 HCWs agreed to participate (response rate = 81%). The majority were male (65.2%), Saudi (87.6%), under 40 years old (74.3%), married (77.4%), with a monthly income of more than 10,000 SAR. Sixty-two (3.0%) of the frontline workers reported a history of COVID-19 diagnosis (Table 1). 

**Table 1:** Sociodemographic characteristics of the participants (n=2094)

	n	%
Gender		
Female	728	34.8
Male	1366	65.2
Nationality		
Saudi	1835	87.6
Non-Saudi	259	12.4
Age group		
<40	1556	74.3
≥40	538	25.7
Marital status		
Married	1621	77.4
Singles	379	18.1
Widowed/divorced	94	4.5
Income		
≤10000	749	35.8
>10000	1345	64.2
COVID-19 infection		
Yes		3.0
No	1395	66.6
I don’t know	637	30.4
Smoking		
Yes	618	29.5
No	1476	70.5
Exercise		
Yes	656	31.3
No	1438	68.7
Mediation		
Yes	526	25.1
No	1568	74.9
Visiting psychiatric clinic last year		
At least one time	171	8.2
No	1923	91.8
Try to reach psychological services last year		
At least one time	136	6.5
No	1958	93.5

Nurses formed the majority of the sample (47.9%), followed by other health specialties (emergency medical services technicians, psychologists, and social worker staff) (26.45%) and doctors (14.7%). Most were hospital frontline workers (63%), followed by primary HCWs (24%), and the smallest proportion was from quarantine facilities (14%) (Table 2).

**Table 2:** Work-related characteristics of the participants (n=2094)

	n	%
Work site		
Hospital	1309	62.5
Primary health care center	493	23.5
Quarantine	292	13.9
Years of experience		
≤7	543	25.9
>7	1551	74.1
Working hours/week		
≤45	631	30.1
>45	1463	69.9
Health care worker category		
Doctors	304	14.7
Nurses	991	47.9
Administrative	227	11.0
Other	572	27.3
Region of practice		
Medina	80	3.8
Najran	316	15.2
North boarders (Hail, Arar, & Aljouf)	195	9.4
Asir (Abha & Albaha)	220	10.6
Eastern province	116	5.6
Riyadh	621	29.9
Qassim	102	4.9
Makkah (Jeddah, Taif, & Alqunfudah)	336	16.2
Hafralbaten	71	3.4
Other	21	1.0

Coping skills and help-seeking behavior

Approximately 31.3% exercised regularly, 29.5% were smokers, and 25.1% reported practicing meditation. Regarding past-year professional mental health service utilization, 8.2% visited the psychiatry clinic at least once over the last year and 6.5% tried to reach psychological services at least once over the previous year.

Perceived sustainability of the stress

Approximately one-quarter of the participants believed that psychological distress was always related to any of the health conditions they may have suffered over the last month (Table 3). Despite the prevalence of psychological distress, about 60% denied their inability to complete either half or all their daily tasks. Regarding the reported daily inability to complete either half or all their daily tasks, the proportion did not exceed 6% of the total responses.

**Table 3:** The perceived stress-related health conditions over the last month

Response	n (%)
A little of the time	391 (19)
Some of the time	205 (10)
Most of the time	1013 (49)
All the time	480 (23)

Level of psychological distress among COVID-19 frontline HCWs

The mean (SD) K10 score was 23.1 (+21). Severe psychological distress was reported by n = 570 (27.3%) of the participants while mild and moderate psychological distress was reported by n = 328 (15.7 %) and n = 277 (13.2%), respectively.

Factors associated with severe psychological distress in univariate analysis

Psychological distress was significantly higher among females (p < 0.001), aged >40 years (p = 0.001), married (p = 0.045), and those who did not reach psychological services last year (p = 0.048). Regarding work-related characteristics, psychological distress was significantly higher among those who worked >45 hours per week (p = 0.010) and those who had more than seven years of experience (p = 0.007). There was an overall significant association between psychological distress and practice region, where the highest reported severe distress was in Asir and the lowest was in the Hafralbaten area (p = 0.005) (Table 4).

**Table 4:** Association between severe psychological distress and sociodemographic variables in univariate analysis (n=2094)

	Severe psychological distress n (%)	P-value
Gender		
Female	157 (21.7)	
Male	413 (30.3)	<0.001
Nationality		
Saudi	504 (27.5)	
Non-Saudi		0.589
Age group		
<40	395 (25.4)	
≥40	176 (32.7)	0.001
Marital status		
Married	23(24.5)	
Singles	463(28.6)	
Widowed/divorced	85(22.4)	0.045
Income		
≤10000	186 (24.8)	
>10000	385 (28.6)	0.062
COVID-19 infection		
Yes	21(33.9)	
No/I don’t know	550(27.1)	0.236
Smoking		
Yes	171 (27.7)	
No	400 (27.1)	0.789
Exercise		
Yes	196 (29.9)	
No	375 (26.1)	0.070
Mediation		
Yes	151 (28.7)	
No	420 (26.8)	0.392
Visiting psychiatric clinic last year		
At least one time	53(31.7)	
No	518(26.9)	0.177
Try to reach psychological services last year		
At least one time	47 (26.8)	
No	524 (34.6)	0.048

Factors associated with severe psychological distress in multivariate analysis

The identified predictors included male gender (OR = 1.5, 95%CI 1.21-1.90), over 40 years old (OR = 1.3, 95%CI 1.01-1.60), working more than 45 hours per week (OR = 1.3, 95%CI 1.07-1.64), with more than seven years of experience (OR = 1.2, 95% CI 1.23-1.53). Furthermore, those who did not try to reach psychological services last year had a higher risk of psychological distress (OR = 1.5, 95% CI 1.03-2.18). As to regional differences, those who worked in Asir had higher distress than those who worked in Hafralbaten (OR = 2.6, 95%CI 1.38-5.05) (Tables 5-6).

**Table 5:** Association between severe psychological distress and work-related variables in univariate analysis (n=570)

	Severe psychological distress n (%)	P-value
Worksite		
Hospital	359 (27.4)	
Primary health care center	125 (25.4)	
Quarantine	87(29.8)	0.393
Years of experience		
≤7	124(22.8)	
>7	447(28.8)	0.007
Working hours/week		
≤45	196(25.6)	
>45	375(31.1)	0.010
Healthcare worker category		
Doctors	84 (27.6)	
Nurses	282 (28.5)	
Administrative	51 (22.5)	
Other	140 (25.6)	0.261
Region of practice		
Medina	23 (28.8)	
Najran	82 (25.9)	
North boarders (Hail, Arar, & Aljouf)	48 (24.6)	
Asir (Abha & Albaha)	87 (39.5)	
Eastern province	31 (26.7)	
Riyadh	164 (26.4)	
Qassim	34 (33.3)	
Makkah (Jeddah, Taif, & Alqunfudah)	81 (24.1)	
Hafralbaten	14 (19.7)	
Other	5 (23.8)	0.005

**Table 6:** Factors associated with severe psychological distress in multivariate analysis

	B	S.E.	P-value	Odds Ratio	95.0% CI
Males	0.420	0.113	0.000	1.5	1.21-1.90
Age ≥40 years	0.243	0.117	0.038	1.3	1.01-1.60
Working hours/week (>45)	0.284	0.108	0.009	1.3	1.07-1.64
Try to reach psychological services last year (no vs yes)	0.406	0.192	0.034	1.5	1.03-2.18
Years of experience (>7 years)	0.182	0.126	0.048	1.2	1.23-1.53
Asir (reference= Hafralbaten)	0.972	0.331	0.003	2.6	1.38-5.05
Constant	-2.092	0.330	0.000	0.12	1.21-1.90

## Discussion

Our study showed that the overall national prevalence of severe psychological distress among all COVID-19 frontline HCWs was 27.3%. Subgroup analysis showed severe psychological distress among male frontline workers. Males suffered a more significant impact of psychological distress than female frontline workers (p = .001). Within this context, a recent review by Bohlken, Schömig, Lemke, Pumberger, and Riedel-Heller on 14 different studies about psychological distress in COVID-19 HCWs reported that the prevalence of psychological distress ranged from 2.2% to 14.5% of all participants [13]. In an extreme finding published by Lai et al. in 2020, nurses, women, frontline HCWs, and those working in Wuhan, China, reported severe degrees of psychological distress, with a prevalence of 71% [12]. The sex differences observed in our study were attributed to the higher representation of male workers, forming 65% of the sample. Moreover, the observed variability in the prevalence of psychological distress across various studies globally can be related to the difference in psychometric tools used and male roles in Middle Eastern society.

Our findings align with those of Balay-Odao et al., who performed a study in Riyadh and concluded that male Saudi national frontline nurses providing direct care for COVID-19 patients were impacted significantly by psychological burden [7]. Moreover, male nurses had a higher mean psychological burden score than female nurses during the 2020 pandemic. The research findings were based on the role of males within Asian families. Likewise, male workers are usually the primary source of income and are the primary providers for the family.

Our study showed a 27.6% prevalence (n = 84) of severe psychological distress among frontline doctors, as shown in Table 6. The findings contradict Almater, Tobaigy, Younis, Alaqeel, and Abouammoh among 107 ophthalmologists in Riyadh, Saudi Arabia, who showed a prevalence of severe depression and anxiety of 3.7% and 5.6%, respectively [14]. The observed higher severity might be due to the broad scope of practice observed for our participating physicians. However, the differences in psychological distress severity among physicians in different specialties at the national level should be assessed in further studies using consistent standardized psychometric tools.

Frontline HCWs working within the KSA who worked for more than 45 hours a week were at the highest risk of severe psychological distress. Several studies have concluded the association between depression and mental distress with long-term weekly working hours [15-16]. However, the results were contradictory based on the country of practice, type of occupation, and working conditions. In the previous study on the average weekly working hours, Kim et al. concluded that for unsecured workers, 41 to 52 hours of working per week correlates with a lower risk of depression and anxiety than working for less than 41 hours per week [15]. From the perspective of COVID-19, a recent study reported by Cai, Tu, Ma, Chen, Fu, Jiang, and Zhuang from Hubei, China, concluded that exhaustion and long working hours during COVID-19 were significantly associated with stress in frontline HCWs [17]. The relation between working hours and occupational health issues is being investigated at the organizational and national levels to resolve the contradictory findings in various studies.

The reported rate of severe psychological distress was directly related to HCW age. At the ages of less than 40 years, the proportion of severe distress was n = 395 (25.4%). Whereas, over age 40, it increased to n = 176 (32.7%). Despite not asking the participants direct questions regarding concerns about infecting family members, more than three-quarters of the participants (n = 1621; 77.4%) were either married with children or married with no children (Table 1). Similarly, Cai et al. concluded that COVID-19 HCWs aged 31-40 years were stressed due to worries about infecting family members [17].

In all of the previously mentioned demographics, male workers, workers who were married, workers aged >40 years, workers who worked for more than 45 hours, workers with seven years of experience, and workers in the Asir regions were likely to be stressed because of the perceived risk of infecting family members. Studies have shown that workload, a history of previous infections, working in isolation words, exposure to and contact with severe cases, and the deaths of other HCWs were each identified as factors contributing to psychological distress [12,18-19].

This study also showed that workers at PHCs were less likely to suffer from severe psychological distress. This result is likely associated with the observed lower infection rates among PHC workers as compared to the 63% reported infection rate among hospital workers. Our findings can be due to exposure to mild cases in PHCs compared to exposure to more severe COVID-19 cases or deaths encountered in hospitals.

Most of the frontline HCWs who reported a high proportion of severe psychological distress denied trying to reach out to any psychological support services (n = 524; 34.6) within the last year. The findings support the notion of the absence of pre-existing psychological distress and/or illnesses. Unironically, the results from a recent study conducted by Ali, Cole, Ahmed, Hamasha, and Panos, in Alabama in the USA found that none of the participating nurses reported ever seeking help from a psychologist as a coping strategy during the current pandemic [20].

Our study found less frequent coping skills, such as exercising and meditation, at 31.3% and 25.5%, respectively. Coping is one of the strategies used to mitigate the impact of stress. A recent study by Shechter, Diaz, Moise, Anstey, Ye, Agarwal, Abdalla, Brodie, Cannone, Chang, Claassen, Cornelius, Derby, Dong, Givens, Hochman, Homma, Kronish, Lee, Manzano, Mayer, McMurry, Moitra, Pham, Rabbani, Rivera, Schwartz, Schwartz, Shapiro, Shaw, Sullivan, Vose, Wasson, Edmondson, and Abdalla reported that physical activity/exercise was the most common coping behavior performed by New York HCWs during the COVID-19 pandemic (59%). Yoga and meditation were employed [18]. They also reported access to psychotherapy services and online self-guided counseling use by 33% of the participating HCWs [21]. Coping strategies, such as HCW training on building resilience to stress, were considered primary prevention by Preti et al. [22].

Our study findings on the low prevalence of physical activity among HCWs can be due to the low physical activity levels within the Saudi community. A systematic review conducted by Al-Hazzaa in 2018 reported that youth and adults are not active enough to meet the recommended guidelines for physical activity [23]. Moreover, in the Al-Jouf region, Banday, Want, Alris, Alrayes, and Alenzi concluded that 35% of PHC physicians were physically inactive [24].

Smoking was a negative coping skill reported by 30% of frontline workers. The lack of physical activity and nicotine dependence should be addressed through public health programs to prevent the long-term effects of sedentary life and substance dependence. Recommended interventions include access to resilience training and online self-help psychological support services to prevent the progression of distress into severe mental illnesses [21-22].

Our findings yield results on the attributes of severe psychological distress encountered by MOH HCWs with COVID-19 within Saudi Arabia. The Kessler psychological distress scale (k10) for measuring anxiety and depression is a reliable scale used in many international public surveys. At the Saudi national level, our study is the first of its kind to assess the prevalence of psychological distress in an occupational setting. Also, none of the previous studies of this kind have included community (primary healthcare and quarantine) and hospital frontline health workers. Multivariate analysis for a relatively large sample has provided adjustment for the potential confounders in this kind of survey. Hence, we may draw a valid conclusion. Moreover, the findings will help identify policy interventions and develop preventive programs. Additionally, the results will help develop coping strategies at both individual and organizational levels.

This study is not a longitudinal cohort study; therefore, there are some limitations such as the risk of missing and/or reporting pre-existing mental health conditions. Additionally, despite a response rate of 81%, response bias may still exist because non-responders were overwhelmed and/or distressed and may not have been interested in filling out the survey. Regional differences and organizational work-related potential stressors, such as workload, payment status, and job satisfaction, were not assessed and are beyond the scope of this study.

## Conclusions

Self-rated severe psychological distress without mental disorder is significantly prevalent among male COVID-19 frontline workers aged >40 years in Saudi Arabia. However, distress did not affect their daily life routines. Hospital frontline workers with a history of infection with severe acute respiratory syndrome coronavirus 2 (SARS-CoV-2) were more likely to report severe psychological distress. Additionally, working an average of more than 45 hours per week was a strong predictor of severe psychological distress. Severe distress was perceived to be highly related to their health conditions and suffering over the last month before responding to the survey.
